# Characteristics and prognostic values of abdominal aortic branches calcification in hemodialysis patients

**DOI:** 10.1080/0886022X.2024.2432538

**Published:** 2025-01-06

**Authors:** Wen Shi, Xiaotong Xie, Yu Zhao, Yuqiu Liu, Xiaoliang Zhang

**Affiliations:** Institute of Nephrology, Zhong Da Hospital, Southeast University School of Medicine, Nanjing, Jiangsu, China

**Keywords:** Hemodialysis, mortality, prognostic model, vascular calcification

## Abstract

**Background:**

Vascular calcification is highly prevalent and associated with mortality in hemodialysis patients. However, extreme splanchnic arterial calcification in calciphylaxis with poor prognosis raises questions regarding the reliability of previous vascular calcification scoring methods. Therefore, this study aimed to examine the distribution characteristics of abdominal aortic branch calcification and identify a more reliable predictor of mortality in hemodialysis patients.

**Methods:**

The cohort study included 237 hemodialysis patients. The distribution characteristics of abdominal aortic branch calcification were determined by quantifying the calcification volumes. The primary and secondary outcomes were all-cause mortality and new-onset cardiovascular events, respectively. We compared the prognostic values of abdominal aortic branch calcification and constructed a predictive nomogram model.

**Results:**

The prevalence of abdominal vascular calcification in hemodialysis patients was 95.36%, with the highest prevalence in the abdominal aorta (88.61%) and internal iliac artery (85.65%). During a median follow-up period of 3.92 years, 137 patients died. Internal iliac artery and mesenteric artery calcification showed the greatest predictive values for mortality. Internal iliac artery calcification and serum albumin level were independently associated with mortality in hemodialysis patients (*p* < .001). The nomogram model constructed with internal iliac artery calcification, serum albumin level, age, and comorbid cardiovascular disease was well discriminative, calibrated, and clinically applicable for predicting 3-year survival.

**Conclusion:**

Abdominal aortic branch calcification, particularly internal iliac artery calcification, is a preferable prognostic predictor than abdominal aorta or coronary artery calcification in hemodialysis patients.

## Introduction

Despite the rapid development of hemodialysis technology, morbidity and mortality remain extremely high in hemodialysis patients [1]. Their leading cause of mortality is cardiovascular disease, with cardiovascular mortality 20–1000 times higher than that in the general population [2]. Therefore, reliable indicators or models that can predict the prognosis should be explored urgently. In addition to traditional risk factors for atherosclerosis such as diabetes and dyslipidemia, hemodialysis patients at risk of cardiovascular events have chronic kidney disease (CKD)-specific factors such as anemia and vascular calcification [3,4].

Numerous studies have established the prognostic values of arterial calcification in hemodialysis patients through coronary artery calcification (CAC) score or a semiquantitative abdominal aortic calcification (AAC) score [5,6]. However, medial calcification specific to patients with end-stage kidney disease (ESKD) differs from intimal calcification which drives atherosclerosis in large arteries [7]. Therefore, it remains unclear whether hemodialysis patients’ calcification status reflected by coronary artery and aorta can serve as prognostic markers.

Calciphylaxis, a rare and fatal syndrome of vascular calcification, is characterized by ischemic damage caused by systemic microvascular medial calcification and intimal hyperplasia [8]. As a particularly severe form of small-to-medium arterial calcification in hemodialysis patients, calciphylaxis has a dire prognosis, with a 1-year mortality rate of 44% [9]. This reveals a potential relationship between medium arterial calcification and prognosis in hemodialysis patients.

Hence, we hypothesized that abdominal aortic branches calcification (AABC) in hemodialysis patients might predict prognosis, with a potentially higher prognostic value than that of AAC or CAC. This cohort study aimed to (1) quantitatively evaluate characteristics of abdominal vascular calcification in hemodialysis patients; (2) compare prognostic values of AAC, AABC, and CAC; (3) construct and validate a prognostic model based on abdominal vascular calcification.

## Methods

### Study participants

We conducted a retrospective cohort study at Southeast University affiliated Zhongda Hospital, and recruited adult patients on regular hemodialysis who had undergone abdominal CT scans between January 2015 and September 2017. Exclusion criteria were as follows: no baseline data, dialysis vintage <3 months, participation in intervention studies, pregnancy or breastfeeding, and diagnosis of life-threatening comorbidities such as malignancy, cirrhosis, heart failure (New York Heart Association functional class III or IV), congenital heart disease, or rheumatic heart disease. The study protocol conformed to the principles of the Declaration of Helsinki. It was approved by the Institutional Ethics Committee for Clinical Research of Southeast University affiliated Zhongda Hospital (2020ZDSYLL218-P01) and registered in the Chinese Clinical Trial Registry (ChiCTR2000040842). Written informed consent was obtained from all the patients.

### Data collection

Baseline demographic, clinical and laboratory data were collected from the hospital information system. The arterial calcification volume was assessed using CT scans and the open-source software 3D Slicer (version 4.11.20210226, RRID: SCR_005619). The arteries measured were the abdominal aorta and its splanchnic branches (celiac trunk, renal artery, mesenteric artery, common iliac artery, and internal iliac artery). Arterial calcification was defined as a region of hyperattenuation >130 Hounsfield units within a 1-mm^2^ area. The marked calcification areas of target arteries in two-dimensional images were integrated into three-dimensional images to calculate the volume (Supplementary Figure 1). Two professionally trained researchers, who were blinded to patients’ identification, independently performed the analysis; the mean was used as the final measurement. In case of discrepancies of >5 mm^3^, a specialist reviewed the results to reach a consensus. CAC volume was assessed in 187 participants who underwent chest CT scans.

### Study outcomes

The observation period began from the date of the CT examination to September 2020. The primary outcome was all-cause mortality, while the secondary outcome was new-onset cardiovascular events including myocardial infarction, heart failure, stroke, malignant arrhythmia, sudden cardiac arrest, peripheral artery disease, and death caused by the abovementioned conditions. The outcome data were collected from the hospital information system.

### Statistical analysis

Missing data with a proportion of <20% were imputed using multiple imputation methods. Normal distribution and homogeneity of variance were assessed by the Shapiro-Wilk test and Levene test, respectively. Continuous variables with a normal distribution were presented as mean and standard deviation (SD), while those with a skewed distribution were presented as median and interquartile range (IQR). For categorical variables, frequency and percentage were used. The arterial calcification volume was categorized according to the median. Differences between groups were compared using two-sample Student’s *t* test, Mann–Whitney *U* test, chi-square test, and Fisher’s exact test.

The cumulative event rate was calculated using the Kaplan–Meier method, and survival differences between groups were analyzed by log-rank test. The prognostic values of arterial calcification volume were assessed using the area under the time-dependent receiver operating characteristic (ROC) curve (AUC). Participants were randomly divided into training and validation datasets in a 7:3 ratio. In the training cohort, the association between arterial calcification and outcome was examined using univariate Cox regression analysis. Arterial calcification volumes were evaluated after a logarithmic transformation. After least absolute shrinkage and selection operator (LASSO) regression, potential covariates would be adjusted in multivariate Cox regression analysis. The hazard ratio (HR) and 95% confidence interval (CI) for arterial calcification were calculated. Subsequently, a nomogram was constructed to predict the prognosis. In both datasets, the discriminative ability, accuracy, and clinical net benefit were evaluated using the time-dependent ROC, calibration curve, and decision curve analysis, respectively. Statistical significance was defined as *p* < .05 in two-tailed tests. Statistical analyses were performed using IBM SPSS Statistics (version 26.0, IBM Corporation, Armonk, NY, USA) and R (version 4.1.2, R Foundation for Statistical Computing, Vienna, Austria) with car, rms, pROC, timeROC, ggDCA, glmnet, VIM, pec, dplyr, nnet and survival packages.

## Results

### Baseline characteristics

**Figure 1.**
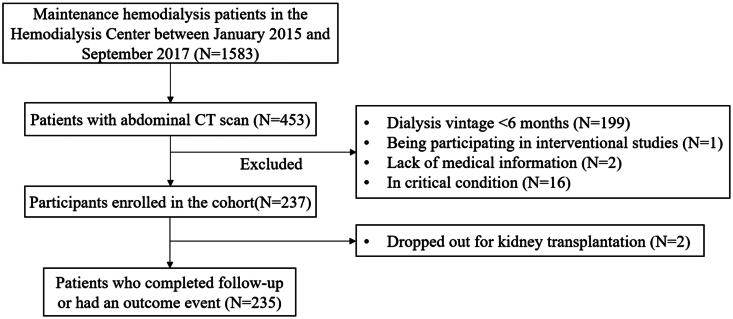
Flow diagram of the cohort study.

Overall, 237 patients were included in the cohort (Figure 1). Baseline data, classified by median of abdominal vascular calcification volume, are shown in Table 1. The median age of patients was 62 (IQR 52–72) years, and 125 (52.74%) participants were men. The median dialysis vintage was 42.00 (IQR 15.50–93.00) months. The severe calcification group was more likely to be older, have a longer dialysis vintage and comorbidities such as cardiovascular disease and atrial fibrillation (*p* < .05).

**Table 1.** Baseline characteristics according to total volume of abdominal vascular calcification.

Variables	Overall (*N* = 237)	Mild calcification (*n* = 118)	Severe calcification (*n* = 119)	*p*-value
Age (years), median (IQR)	62.00 (52.00,74.00)	54.00 (46.75,63.00)	71.00 (61.00,79.00)	<.001
Male, *n* (%)	125 (52.74)	63 (53.39)	62 (52.10)	.842
Dialysis vintage (months), median (IQR)	39.00 (14.00,92.00)	29.50 (12.00,69.75)	48.00 (20.00,108.00)	.002
BMI, *n* (%)				.130
<18.50 kg/m^2^	26 (10.97)	11 (9.32)	15 (12.61)	
18.50–23.99 kg/m^2^	125 (52.74)	70 (59.32)	55 (46.22)	
≥24.00 kg/m^2^	86 (36.29)	37 (31.36)	49 (41.18)	
BSA (m^2^), median (IQR)	1.63 (1.49,1.78)	1.65(1.53,1.77)	1.62 (1.47,1.78)	.276
SBP (mmHg), median (IQR)	140.00 (125.00,160.00)	140.00 (129.25,160.00)	142.00 (122.00,160.00)	.920
DBP (mmHg), median (IQR)	80.00 (70.00,86.00)	80.00 (70.00,89.25)	80.00 (70.00,84.00)	.072
Smoking, *n* (%)	27 (11.39)	10 (8.47)	17 (14.29)	.159
Comorbidities, *n* (%)				
Hypertension	210 (88.61)	107 (90.68)	103 (86.55)	.318
Diabetes	90 (37.97)	40 (33.90)	50 (42.02)	.198
Cardiovascular disease	118 (49.79)	43 (36.44)	75 (63.03)	<.001
Atrial fibrillation	34 (14.35)	10 (8.47)	24 (20.17)	.010
Parathyroidectomy, *n* (%)	11 (4.64)	4 (3.39)	7 (5.88)	.362
Medications, *n* (%)				
Vitamin D and analogue	98 (41.35)	45 (38.14)	53 (44.54)	.317
Calcium-based phosphate binder	52 (21.94)	25 (21.19)	27 (22.69)	.780
Calcimimetic	3 (1.27)	2 (1.69)	1 (0.84)	.994
ACEI/ARB	52 (21.94)	26 (22.03)	26 (21.85)	.973
β-blocker	97 (40.93)	50 (42.37)	47 (39.50)	.652
Calcium channel blocker	141 (59.49)	73 (61.86)	68 (57.14)	.459
Warfarin	6 (2.53)	2 (1.69)	4 (3.36)	.687
Antiplatelet agent	41 (17.30)	15 (12.71)	26 (21.85)	.063
Nitrate	76 (32.07)	32 (27.12)	44 (36.97)	.104
Statin	32 (13.50)	15 (12.71)	17 (14.29)	.723
Hemoglobin (g/L), mean (SD)	99.84 ± 23.93	97.35 ± 23.28	102.30 ± 24.41	.124
CRP (mg/L), median (IQR)	23.50 (6.09,77.90)	31.10 (7.25,87.42)	17.60 (5.88,60.20)	.139
Albumin (g/L), median (IQR)	36.50 (33.00,39.70)	36.55 (33.15,40.00)	36.10 (32.80,39.20)	.441
Alkaline phosphatase (U/L), median (IQR)	77.00 (60.00,122.00)	71.50 (58.00,118.00)	82.00 (62.00,123.00)	.173
Glucose (mmol/L), median (IQR)	5.97 (4.82,8.58)	6.01 (4.81,8.32)	5.97 (4.82,8.78)	.877
Creatinine (μmol/L), median (IQR)	673.00 (522.50,887.00)	710.00 (527.00,934.50)	654.00 (508.00,806.00)	.086
Uric acid (μmol/L), median (IQR)	353.00 (274.00,438.00)	368.00 (273.50,454.00)	346.00 (274.00,418.00)	.433
Triglyceride (mmol/L), median (IQR)	1.56 (1.00,2.33)	1.72 (1.07,2.35)	1.45 (0.95,2.21)	.226
Total cholesterol (mmol/L), median (IQR)	3.77 (3.20,4.55)	3.72 (3.19,4.46)	3.80 (3.19,4.64)	.357
HDL (mmol/L), median (IQR)	1.01 (0.81,1.19)	0.98(0.79,1.13)	1.02 (0.83,1.24)	.186
LDL (mmol/L), median (IQR)	2.16 (1.65,2.77)	2.10 (1.67,2.73)	2.22 (1.65,2.79)	.485
Potassium (mmol/L), median (IQR)	4.31 (3.89,4.86)	4.33 (3.87,4.94)	4.28 (3.89,4.83)	.300
Corrected calcium (mmol/L), median (IQR)	2.34 (2.20,2.48)	2.34 (2.21,2.46)	2.33 (2.20,2.49)	.820
Phosphorus (mmol/L), median (IQR)	1.71 (1.23,2.19)	1.78 (1.25,2.18)	1.62 (1.22,2.20)	.635
Ferritin (μg/L), median (IQR)	230.30 (72.00,488.60)	238.30 (85.55,534.91)	224.50 (59.60,447.80)	.296
PTH (pg/ml), median (IQR)	164.30 (72.25,417.82)	151.65 (65.58,477.33)	173.50 (81.00,356.80)	.689
Arterial calcification volume (mm^3^), median (IQR)				
Abdominal aorta	764.82 (47.09,3018.17)	50.30 (0.18,444.19)	3001.95 (1703.69,5235.07)	<.001
Celiac trunk	11.12 (0,202.68)	0 (0,6.84)	135.33 (16.13,627.44)	<.001
Mesenteric arteries	3.05 (0,104.51)	0 (0,2.50)	64.95 (4.65,355.73)	<.001
Renal artery	18.23 (0,135.95)	0.02 (0,21.34)	93.52 (11.59,396.60)	<.001
Common iliac artery	409.37 (3.54,1560.12)	5.72 (0,280.78)	1556.26 (680.83,2781.15)	<.001
Internal iliac artery	235.11 (12.91,925.02)	17.73 (0,154.56)	876.35 (290.43,1849.68)	<.001
All branches	1078.46 (154.66,3446.06)	202.69 (8.50,723.44)	3376.15 (1800.20,6807.82)	<.001
Total	2403.20 (396.08,7010.39)	296.08 (44.99,1141.58)	6959.58 (3511.35,13075.31)	<.001

IQR, interquartile range; SD, standard deviation; BMI, body mass index; BSA, body surface area; SBP, systolic blood pres­sure; DBP, diastolic blood pressure; ACEI, angiotensin-converting enzyme inhibitor; ARB, angiotensin receptor blocker; CRP, C-reactive protein; HDL, high density lipopro­tein; LDL, low density lipoprotein; PTH, parathyroid hormone.

### Characteristics of abdominal vascular calcification

The abdominal aorta and common iliac artery had the largest calcification volumes. Calcification in various arteries differed significantly between the mild and severe calcification groups (*p* < .001; Table 1). The prevalence of abdominal vascular calcification was 95.36%. In particular, AABC (94.51%) was more prevalent than AAC (88.61%). Among the AABC, the internal iliac artery calcification (IIAC) was the most prevalent (85.65%) (Supplementary Table 1).

### Survival analysis

During a median follow-up period of 3.92 years (607 person-years), 137 (57.81%) patients died and 2 patients dropped out for kidney transplantation. The median survival time was 37.00 (95%CI 29.05–44.95) months. Cardiovascular disease was the leading cause of death (54.01%; Supplementary Figure 2). The 1-, 3-, and 5-year cumulative mortality rates were 28.69%, 49.99%, and 64.57%, respectively.

The mild calcification group had a lower cumulative incidence of death (*p* < .001) and longer median survival time (Supplementary Figure 3a). However, there was no statistical difference in death causes between the groups (*p* > .05; Supplementary Table 2). The survival of patients with different degrees of abdominal aorta, celiac trunk, mesenteric artery, and internal iliac artery calcification demonstrated a significant difference (*p* < .001), whereas the extent of renal artery calcification did not impact patients’ prognosis (*p* = .293).

### Prognostic values of abdominal vascular calcification and coronary artery calcification

**Figure 2.**
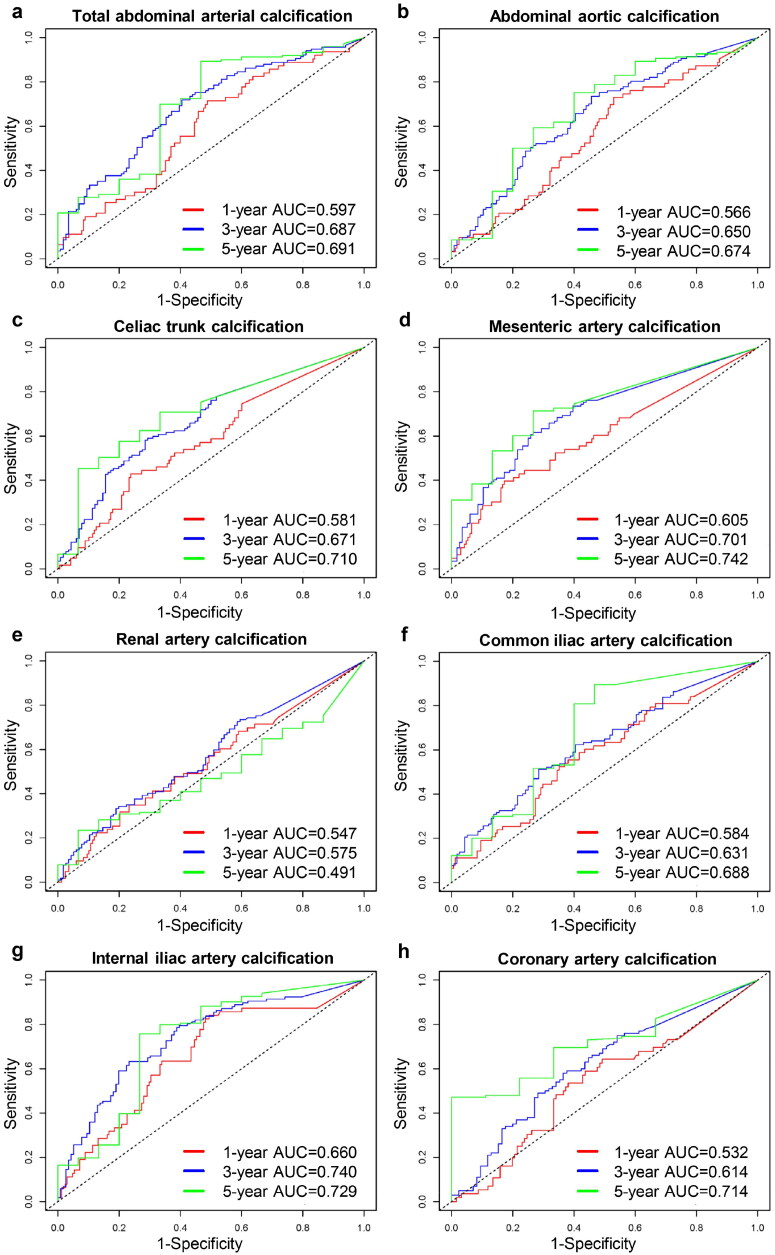
Time-dependent ROC curves of abdominal vascular calcification and coronary artery calcification for predicting survival.

Time-dependent ROC curves illustrating predictive values of arterial calcification for all-cause mortality are presented in Figure 2. The AUC of IIAC for 1- and 3-year survival were the largest, while mesenteric artery calcification had the best predictive value for 5-year survival. Thus, internal iliac artery and mesenteric artery calcifications could potentially be predictors of mortality. Additionally, the predictive value of CAC is not superior to that of AABC. Regarding prediction time, calcification in all arteries except the renal artery had a higher predictive value for 3- and 5-year survival than for 1-year survival.

### Construction, evaluation, and validation of survival prediction model

**Figure 3.**
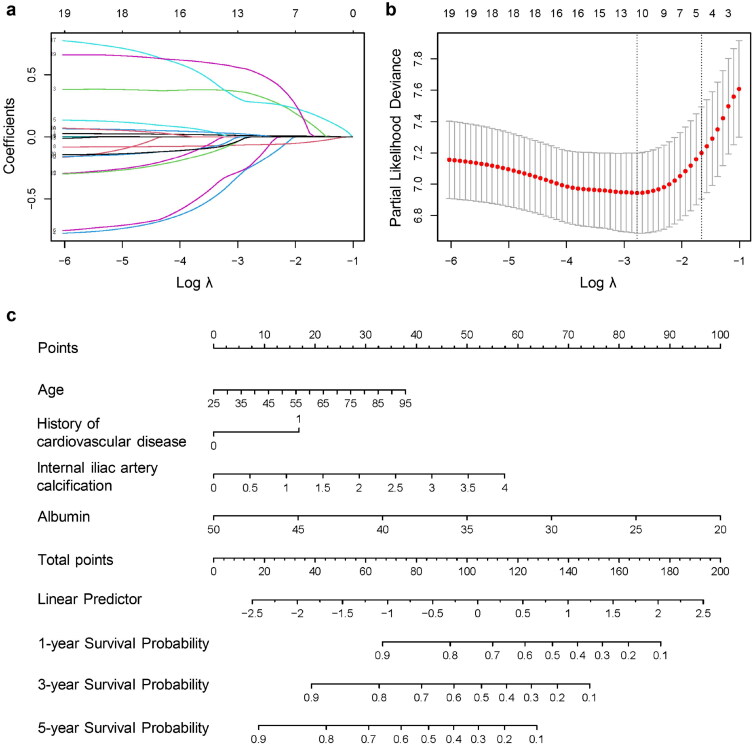
Least absolute shrinkage and selection operator (LASSO) regression analysis and nomogram for predicting survival in hemodialysis patients. (a) LASSO coefficient profile plot of 18 variables. (b) The profile of log(λ) and partial likelihood deviance to identify the optimal penalty coefficient using 10-fold cross-validation and minimum criterion. (c) The nomogram predicting survival of hemodialysis patients based on Cox regression analysis.

The baseline characteristics of patients in the training and validation sets were similar (*p* > .05; Table 2). In the training cohort, univariate Cox regression analysis showed 18 variables were associated with mortality (*p* < .05; Table 3). Considering the potential correlations among these 18 variables, LASSO-Cox regression analysis was utilized to mitigate bias. All 18 variables were included in the LASSO-Cox regression analysis, and the coefficient profile is shown in Figure 3a. Four variables with nonzero coefficients were selected at the optimal penalty coefficient *λ* = 0.19015 to construct the most regularized and parsimonious model (Figure 3b).

**Table 2.** Baseline data of patients in the training cohort and validation cohort.

Variables	Training cohort (*n* = 165)	Validation cohort (*n* = 72)	*p* value
Age (year), median (IQR)	63.00 (53.00,74.00)	60.50 (51.25,74.75)	.334
Male, *n* (%)	92 (55.76)	33 (45.83)	.842
Dialysis vintage (month), median (IQR)	42.00 (14.00,96.00)	37.00 (16.25,81.00)	.081
BMI, *n* (%)			.443
<18.50 kg/m^2^	20 (12.12)	6 (8.33)	
18.50–23.99 kg/m^2^	89 (53.94)	36 (50.00)	
≥24.00 kg/m^2^	56 (33.94)	30 (41.67)	
BSA (m^2^), median (IQR)	1.62 (1.49,1.79)	1.64 (1.49,1.75)	.276
SBP (mmHg), median (IQR)	142.00 (126.00,159.00)	139.00 (124.25,160.00)	.731
DBP (mmHg), median (IQR)	80.00 (70.00,86.50)	80.00 (70.00,84.00)	.496
Smoking, *n* (%)	18 (10.91)	9 (12.50)	.723
Comorbidities, *n* (%)			
Hypertension	148 (89.70)	62 (86.11)	.424
Diabetes	64 (38.79)	26 (36.11)	.696
Cardiovascular disease	86 (52.12)	32 (44.44)	.277
Atrial fibrillation	25 (15.15)	9 (12.50)	.592
Parathyroidectomy, *n* (%)	7 (4.24)	4 (5.56)	.659
Medications, *n* (%)			
Vitamin D and analogue	68 (41.21)	30 (41.67)	.948
Calcium-based phosphate binder	36 (21.82)	16 (22.22)	.945
Calcimimetic	3 (1.82)	0 (0)	.555
ACEI/ARB	32 (19.39)	20 (27.78)	.151
β-blocker	64 (38.79)	33 (45.83)	.310
Calcium channel blocker	94 (56.97)	47 (65.28)	.231
Warfarin	3 (1.82)	3 (4.17)	.543
Antiplatelet agent	29 (17.58)	12 (16.67)	.865
Nitrate	51 (30.91)	25 (34.72)	.563
Statin	23 (13.94)	9 (12.50)	.766
Hemoglobin (g/L), mean ± SD	100.71 ± 24.37	97.83 ± 22.95	.396
CRP (mg/L), median (IQR)	21.80 (5.76,66.00)	23.55 (8.84,90.75)	.616
Albumin (g/L), median (IQR)	36.60 (33.10,40.00)	36.00 (32.75,38.23)	.636
Alkaline phosphatase (U/L), median (IQR)	74.00(59.50,117.00)	83.00 (60.50,126.25)	.671
Glucose (mmol/L), median (IQR)	6.11 (4.84,8.78)	5.79 (4.81,8.22)	.393
Creatinine (μmol/L), median (IQR)	674.00 (526.00,887.00)	672.50 (507.25,922.25)	.861
Uric acid (μmol/L), median (IQR)	359.00 (274.50,419.50)	346.00 (272.25,469.75)	.840
Triglyceride (mmol/L), median (IQR)	1.59 (1.03,2.40)	1.43 (0.96,2.04)	.838
Total cholesterol (mmol/L), median (IQR)	3.83 (3.24,4.57)	3.60 (3.06,4.52)	.712
HDL (mmol/L), median (IQR)	0.98 (0.81,1.18)	1.04 (0.81,1.23)	.936
LDL (mmol/L), median (IQR)	2.19 (1.66,2.77)	2.09 (1.63,2.77)	.709
Potassium (mmol/L), median (IQR)	4.32 (3.90,4.91)	4.25 (3.85,4.81)	.586
Corrected calcium (mmol/L), median (IQR)	2.33 (2.20,2.48)	2.36 (2.22,2.47)	.600
Phosphorus (mmol/L), median (IQR)	1.65 (1.23,2.16)	1.79 (1.25,2.22)	.641
Ferritin (μg/L), median (IQR)	237.20 (76.95,529.98)	186.65 (58.55,422.19)	.855
PTH (pg/ml), median (IQR)	173.00 (81.25,430.09)	126.30 (51.33,326.43)	.775
Arterial calcification volume (mm^3^), median (IQR)			
Abdominal aorta	1188.06 (47.09,3252.61)	694.56 (47.89,2351.46)	.414
Celiac trunk	15.99 (0,223.80)	1.78 (0,164.57)	.754
Mesenteric arteries	2.51 (0,81.20)	9.74 (0,133.91)	.548
Renal artery	19.03 (0,123.33)	16.53 (0,186.07)	.741
Common iliac artery	375.32 (5.72,1613.01)	495.59 (0.39,1114.71)	.275
Internal iliac artery	231.49 (12.91,998.65)	237.13 (11.79,779.12)	.472

IQR, interquartile range; SD, standard deviation; BMI, body mass index; BSA, body surface area; SBP, systolic blood pres­sure; DBP, diastolic blood pressure; ACEI, angiotensin-converting enzyme inhibitor; ARB, angiotensin receptor blocker; CRP, C-reactive protein; HDL, high density lipopro­tein; LDL, low density lipoprotein; PTH, parathyroid hormone.

**Table 3.** Univariate and multivariate Cox regression analysis assessing variables associated with survival in hemodialysis patients.

Variables	Univariate analysis			Multivariate analysis		
	HR	95%CI	*p*-value	HR	95%CI	*p*-value
Age (year)	1.04	1.03–1.06	<.001	1.02	1.00–1.04	.127
Male	0.86	0.58–1.28	.456	–	–	–
Dialysis vintage (month)	1.00	1.00–1.00	.304	–	–	–
BMI (kg/m^2^)			.001			–
<18.50	2.46	1.41–4.29	.001	–	–	–
18.50–23.99	Reference			–	–	–
≥24.00	0.84	0.54–1.31	.452	–	–	–
BSA (m^2^)	0.20	0.07–0.57	.003	–	–	–
SBP (mmHg)	1.00	0.99–1.01	.624	–	–	–
DBP (mmHg)	0.98	0.96–0.99	.007	–	–	–
Smoking	1.58	0.88–2.82	.127	–	–	–
Comorbidities						
Hypertension	0.70	0.38–1.28	.242	–	–	–
Diabetes	1.57	1.06–2.33	.025	–	–	–
Cardiovascular disease	2.78	1.82–4.25	<.001	1.58	0.97–2.59	.069
Atrial fibrillation	1.04	0.62–1.76	.872	–	–	–
Parathyroidectomy	0.18	0.02–1.26	.083	–	–	–
Medications						
Vitamin D and analogue	0.97	0.65–1.45	.898	–	–	–
Calcium-based phosphate binder	0.74	0.45–1.23	.245	–	–	–
Calcimimetic	0.40	0.06–2.90	.367	–	–	–
ACEI/ARB	0.52	0.30–0.92	.025	–	–	–
β-blocker	0.67	0.44–1.03	.066	–	–	–
Calcium channel blocker	0.86	0.58–1.27	.442	–	–	–
Warfarin	0.54	0.08–3.85	.535	–	–	–
Antiplatelet agent	1.00	0.60–1.67	.995	–	–	–
Nitrate	1.59	1.06–2.39	.025	–	–	–
Statin	1.30	0.76–2.22	.340	–	–	–
Hemoglobin (g/L)	1.00	0.99–1.00	.327	–	–	–
CRP (mg/L)	1.00	1.00–1.01	.083	–	–	–
Albumin (g/L)	0.89	0.86–0.93	<.001	0.91	0.88–0.95	<.001
Alkaline phosphatase (U/L)	1.00	1.00–1.01	<.001	–	–	–
Glucose (mmol/L)	1.06	1.02–1.108	.007	–	–	–
Creatinine (μmol/L)	1.00	1.00–1.00	<.001	–	–	–
Uric acid (μmol/L)	1.00	1.00–1.00	.863	–	–	–
Triglyceride (mmol/L)	1.08	0.89–1.31	.456	–	–	–
Total cholesterol (mmol/L)	1.09	0.91–1.29	.345	–	–	–
HDL (mmol/L)	1.51	1.74–3.06	.260	–	–	–
LDL (mmol/L)	1.01	0.81–1.26	.921	–	–	–
Potassium (mmol/L)	0.78	0.59–1.04	.086	–	–	–
Corrected calcium (mmol/L)	0.60	0.26–1.41	.243	–	–	–
Phosphorus (mmol/L)	0.85	0.64–1.14	.278	–	–	–
Ferritin (μg/L)	1.00	1.00–1.00	.614	–	–	–
PTH (pg/ml)	1.00	1.00–1.00	.407	–	–	–
Arterial calcification volume (per 1SD log)						
Abdominal aorta	1.40	1.17–1.67	<.001	–	–	–
Celiac trunk	1.37	1.17–1.60	<.001	–	–	–
Mesenteric arteries	1.60	1.34–1.91	<.001	–	–	–
Renal artery	1.29	1.07–1.55	.008	–	–	–
Common iliac artery	1.31	1.11–1.53	.001	–	–	–
Internal iliac artery	1.78	1.46–2.18	<.001	1.49	1.20–1.85	<.001

BMI: body mass index; BSA: body surface area; SBP: systolic blood pressure; DBP: diastolic blood pressure; ACEI: angiotensin-converting enzyme inhibitor; ARB: angiotensin receptor blocker; CRP: C-reactive protein; HDL: high density lipoprotein; LDL: low density lipoprotein; PTH: parathyroid hormone.

Multivariate analysis was then performed to adjust for covariates (Table 3). IIAC (HR 1.49, 95%CI 1.20–1.85, *p* < .001) and serum albumin level (HR 0.91, 95%CI 0.88–0.95, *p* < .001) was independently associated with mortality. The nomogram predicting the prognosis included IIAC, serum albumin level, age, and comorbid cardiovascular disease (Figure 3c).

**Figure 4.**
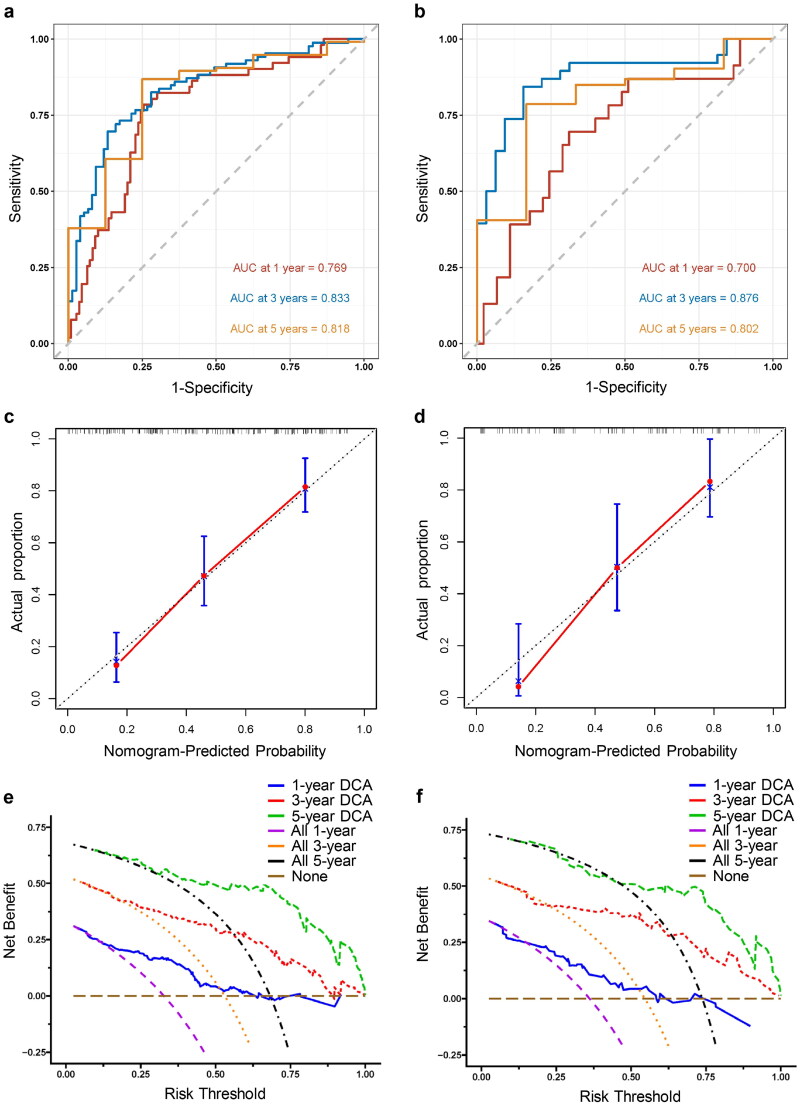
Evaluation and validation of the prognosis model. Time-dependent ROC curves of the model in the training (a) and validation (b) cohort. Calibration curves of the model predicting 3-year survival in the training (c) and validation (d) cohort by 1000-time bootstrap resampling. Decision curves of the model in the training (e) and validation (f) cohort.

The C-index of the model were 0.747 (95%CI 0.722–0.772) and 0.753 (95%CI 0.715–0.791) in the training and validation sets, respectively. Time-dependent ROC curves exhibited excellent predictive values for 3- and 5-year survival, with AUC >0.800 in both sets (Figure 4a,b). Calibration curves after bootstrap resampling showed good conformance between actual and predicted probability in both datasets (Figure 4c,d). The clinical application value of the model for predicting the 3-year survival was higher than that for 1- or 5-year survival (Figure 4e,f).

### Secondary outcome analysis

Of 237 patients, 122 (51.48%) experienced new-onset cardiovascular events, with a cumulative incidence of 31.71%, 57.89%, and 91.73% at 1, 3, and 5 years, respectively. The mild calcification group has a lower cumulative incidence of cardiovascular events (*p* = .012; Supplementary Figure 3b). The AUC showed that IIAC and mesenteric artery calcification had the best predictive values for cardiovascular events (Supplementary Figure 4). Although CAC exhibited a slightly better predictive value for 3-year cardiovascular events than IIAC, it had low predictive values for 1- and 5-year events. IIAC (HR 1.27, 95% CI 1.07–1.52, *p* = .006) and comorbid cardiovascular disease (HR 1.73, 95% CI 1.18–2.54, *p* = .005) were identified as independent risk factors for new-onset cardiovascular events.

### Subgroup analysis

Given the high prevalence of medial calcification in diabetic patients, we analyzed 90 hemodialysis patients with diabetes. The prognosis was significantly poorer in patients with diabetes than in those without (median survival time 21.00 vs 48.00 months; *p* = .001). The LASSO-Cox regression analysis revealed IIAC as the strongest predictor of prognosis. An increase of per SD in the logarithmic volume of IIAC was associated with a 1.79-fold increase in mortality risk (*p* < .001). In addition, we analyzed prognostic predictors in 147 hemodialysis patients without diabetes, and found that IIAC was also an independent risk factor (HR 1.39, 95% CI 1.10–1.76, *p* = .006; Supplementary Table 3).

## Discussion

This cohort study investigated the distribution of abdominal vascular calcification and compared prognostic values of different calcification areas in hemodialysis patients. The prevalence of abdominal vascular calcification was high, with AAC and IIAC being the most prevalent. Notably, IIAC was identified as a superior predictor compared with other vascular calcification, and as an independent risk factor for mortality. Furthermore, a nomogram model based on IIAC was developed and validated, demonstrating a good predictive value for prognostication.

Vascular calcification can be divided into intimal and medial calcifications. Intimal calcification causes atherosclerosis, typically involving large- and medium-sized arteries [10]. CKD is considered to be a promoting factor rather than an initiating factor for intimal calcification. Medial calcification is similar to physiological bone formation, and it results from the conversion of vascular smooth muscle cells into osteo/chondrocyte-like cells [11]. It is distributed along elastic fibers, leading to arterial stiffness and hemodynamic disturbances [12]. Medial calcification is prevalent in patients with CKD and diabetes, affecting small- and medium-sized vessels, and presents as systemic calcification with or without intimal atherosclerosis [7,12,13]. Medial calcification of iliac arteries in dialysis patients has been identified using various imaging and pathological techniques [14]. In our study, hemodialysis patients exhibited a remarkably high prevalence of vascular calcification in the abdominal aorta and its splanchnic branches, suggesting a propensity for both intimal and medial calcifications, although it is difficult to distinguish the two types of calcifications by CT. The prevalence of arterial calcification in hemodialysis patients reportedly ranges 56.5%–77.8% using the AAC scoring method [5,15]; this rate is lower than our results. The discrepancy can be attributed to several reasons. First, our study comprehensively evaluated abdominal vascular calcification, rather than AAC alone, indicating that the previous AAC score underestimated hemodialysis patients’ calcification. Second, vascular calcification was assessed objectively using calcification volume, which was distinct from the previous semiquantitative measures of visual inspection [16]. Finally, hemodialysis patients may exhibit a higher prevalence of vascular calcification because of various mechanisms, such as mineral metabolism disorders and oxidative stress [7]. Our participants were characterized by advanced age, long dialysis vintage, and a high burden of comorbidities, indicative of a more vulnerable population than those in previous studies.

Vascular calcification is a critical contributor to mortality and cardiovascular events in hemodialysis patients [17]. AAC and CAC, associated with atherosclerosis, independently increase mortality risk [6,18]. Medial calcification triggers arterial stiffening and correlates with cardiovascular mortality, lower limb ischemia, and amputation [19]. A 3-year prospective cohort study showed that rather than AAC score, Adragao score evaluating arterial calcification in the pelvis and hands, is an independent risk factor for all-cause and cardiovascular mortality in CKD patients [20]. Calciphylaxis, which often manifests as an extremity gangrene, is a typical example of medial calcification and a risk factor for mortality in hemodialysis patients [21,22]. Niu et al. [23] found that only AAC and femoral artery calcification were independent predictors of mortality excluding iliac artery and radial artery calcification, with AAC being more predictive. We verified that vascular calcification is associated with all-cause mortality and cardiovascular events in hemodialysis patients. Notably, IIAC exhibited a superior predictive value compared with AAC. The disparity may be attributed to dissimilarities in the abovementioned vascular calcification scorings and various statistical methods. However, femoral artery calcification was not evaluated; thus, we failed to compare predictive values of femoral artery calcification and IIAC. Medial calcification was also a stronger predictor of cardiovascular mortality than intima calcification in diabetics [24]. We confirmed the prognostic value of IIAC in hemodialysis patients with diabetes, suggesting the influence of medial calcification. The Kidney Disease Improving Global Outcomes (KDIGO) CKD-MBD Work Group recommends regular vascular calcification assessment through radiology in hemodialysis patients [25]. However, in addition to assessing intimal calcification, such as CAC and AAC, we believe that evaluating medial calcification is equally, if not more, important.

Nutritional status is also a crucial factor that influences the prognosis of hemodialysis patients, who are susceptible to protein-energy wasting (PEW). PEW refers to malnutrition caused by dietary restrictions, anorexia, and wasting conditions resulting from inflammation [26,27]. PEW, the prevalence of which is 28%–54% in hemodialysis patients, decreases their quality of life and increases mortality [28]. Additionally, it triggers heart failure and infections, creating a vicious cycle that negatively affects the prognosis [29]. Serum albumin level, BMI (body mass index), and serum creatinine levels are indicators of PEW [26]. Our study revealed that hypoalbuminemia was an independent risk factor for mortality, consistent with previous studies [30]. In the univariate analysis, BMI <18.50 kg/m^2^ was associated with increased mortality, known as the ‘obesity paradox’ [31]. As noted by Su et al. [32], the optimal BMI for hemodialysis patients requires further investigation because of potential misclassification and selection bias in previous studies. Serum creatinine level, indicating muscle mass and meat intake, is a marker of sarcopenia related to PEW [33] and was associated with mortality in the univariate analysis. Hence, nutritional status assessment and nutritional supplementation are important for the prognosis of hemodialysis patients. Additionally, among single nutrition-related indicators, serum albumin had the largest impact on the prognosis.

The leading cause of mortality in hemodialysis patients was cardiovascular disease, and comorbid cardiovascular disease was included in the prognostic model. This suggested that irreversible cardiovascular damage might escalate the risk of recurrent cardiovascular events. A prospective cohort study has demonstrated that hemodialysis patients with a history of cardiovascular disease have a 1.68–2.11 times greater risk of recurrent cardiovascular events [34]. Nitrate use was a perplexing risk factor for mortality in univariate analysis. Actually, it can be explained by medical history, as patients’ cardiovascular damage may be difficult to reverse with nitrates.

The study has some strengths. It provides a comprehensive and quantitative assessment of abdominal aortic branches calcification in hemodialysis patients, employing a more detailed and objective scoring method than previous approaches. It also compares the prognostic value of abdominal aortic and its branches calcification utilizing a variety of statistical analysis methods such as time-dependent ROC analysis and LASSO regression analysis. Furthermore, long-term follow-up with low rate of loss and comprehensive statistical methods ensured the authenticity and reliability.

There are some limitations in our study. Firstly, as a retrospective cohort study conducted in a tertiary hospital, it is susceptible to information and selection bias. To overcome it, a multicenter prospective cohort study is currently underway. Additionally, given the limited coverage of abdominal CT, we did not evaluate calcification of lower-extremity arteries, such as the femoral artery, so we could not identify their prognostic values. However, the prognostic value of IIAC already reveals a propensity. Lastly, dynamic changes in vascular calcification may have a greater predictive value for long-term prognosis; hence, they warrant further exploration.

In conclusion, IIAC had a better prognostic value for hemodialysis patients than CAC and AAC. This obliges us to shift the focus of vascular calcification assessment from coronary artery and abdominal aorta to splanchnic branches and peripheral arteries that are susceptible to medial calcification in hemodialysis patients. Further validation is necessary to extend the application of the vascular calcification-based nomogram model to ESKD or even CKD populations. Hemodynamic and basic studies are required to elucidate mechanisms underlying vascular calcification. We were inspired by a rare disease to study common diseases in vulnerable populations, reflecting the significance of rare disease research and providing perspectives for future research on common diseases to benefit more patients.

## Supplementary Material

revised_Supplementary_materials new.docx

## Data Availability

The data involved in this article are available from the corresponding author on reasonable request.

## References

[1] Saran R, Robinson B, Abbott KC, et al. Us renal data system 2019 annual data report: epidemiology of kidney disease in the United States. Am J Kidney Dis. 2020;75(1 Suppl 1):A6–A7. doi: 10.1053/j.ajkd.2019.09.003. 31704083

[2] Ortiz A, Covic A, Fliser D, et al. Epidemiology, contributors to, and clinical trials of mortality risk in chronic kidney failure. Lancet. 2014;383(9931):1831–1843. doi: 10.1016/S0140-6736(14)60384-6. 24856028

[3] Baber U, de Lemos JA, Khera A, et al. Non-traditional risk factors predict coronary calcification in chronic kidney disease in a population-based cohort. Kidney Int. 2008;73(5):615–621. doi: 10.1038/sj.ki.5002716. 18075501

[4] Dilsizian V, Gewirtz H, Marwick TH, et al. Cardiac imaging for coronary heart disease risk stratification in chronic kidney disease. JACC Cardiovasc Imaging. 2021;14(3):669–682. doi: 10.1016/j.jcmg.2020.05.035. 32828780

[5] Inoue H, Shimizu S, Watanabe K, et al. Impact of trajectories of abdominal aortic calcification over 2 years on subsequent mortality: a 10-year longitudinal study. Nephrol Dial Transplant. 2018;33(4):676–683. doi: 10.1093/ndt/gfx253. 28992124

[6] Bellasi A, Di Lullo L, Russo D, et al. Vascular calcification progression modulates the risk associated with vascular calcification burden in incident to dialysis patients. Cells. 2021;10(5):1091. doi: 10.3390/cells10051091. 34063597 PMC8147653

[7] Nelson AJ, Raggi P, Wolf M, et al. Targeting vascular calcification in chronic kidney disease. JACC Basic Transl Sci. 2020;5(4):398–412. doi: 10.1016/j.jacbts.2020.02.002. 32368697 PMC7188874

[8] Nigwekar SU, Thadhani R, Brandenburg VM. Calciphylaxis. N Engl J Med. 2018;378(18):1704–1714. doi: 10.1056/NEJMra1505292. 29719190

[9] Gabel CK, Nguyen ED, Chakrala T, et al. Assessment of outcomes of calciphylaxis. J Am Acad Dermatol. 2021;85(4):1057–1064. doi: 10.1016/j.jaad.2020.10.067. 33130181

[10] Bernelot Moens SJ, Verweij SL, van der Valk FM, et al. Arterial and cellular inflammation in patients with CKD. J Am Soc Nephrol. 2017;28(4):1278–1285. doi: 10.1681/ASN.2016030317. 27799487 PMC5373444

[11] Voelkl J, Lang F, Eckardt K, et al. Signaling pathways involved in vascular smooth muscle cell calcification during hyperphosphatemia. Cell Mol Life Sci. 2019;76(11):2077–2091. doi: 10.1007/s00018-019-03054-z. 30887097 PMC6502780

[12] Hénaut L, Chillon J-M, Kamel S, et al. Updates on the mechanisms and the care of cardiovascular calcification in chronic kidney disease. Semin Nephrol. 2018;38(3):233–250. doi: 10.1016/j.semnephrol.2018.02.004. 29753400

[13] Manzoor S, Ahmed S, Ali A, et al. Progression of medial arterial calcification in CKD. Kidney Int Rep. 2018;3(6):1328–1335. doi: 10.1016/j.ekir.2018.07.011. 30450459 PMC6224661

[14] Schlieper G, Aretz A, Verberckmoes SC, et al. Ultrastructural analysis of vascular calcifications in uremia. J Am Soc Nephrol. 2010;21(4):689–696. doi: 10.1681/ASN.2009080829. 20203159 PMC2844300

[15] Kraus MA, Kalra PA, Hunter J, et al. The prevalence of vascular calcification in patients with end-stage renal disease on hemodialysis: a cross-sectional observational study. Ther Adv Chronic Dis. 2015;6(3):84–96. doi: 10.1177/2040622315578654. 25984289 PMC4416967

[16] Kauppila LI, Polak JF, Cupples LA, et al. New indices to classify location, severity and progression of calcific lesions in the abdominal aorta: a 25-year follow-up study. Atherosclerosis. 1997;132(2):245–250. doi: 10.1016/s0021-9150(97)00106-8. 9242971

[17] Reiss AB, Miyawaki N, Moon J, et al. CKD, arterial calcification, atherosclerosis and bone health: inter-relationships and controversies. Atherosclerosis. 2018;278:49–59. doi: 10.1016/j.atherosclerosis.2018.08.046. 30253289

[18] Bellasi A, Ferramosca E, Ratti C, et al. The density of calcified plaques and the volume of calcium predict mortality in hemodialysis patients. Atherosclerosis. 2016;250:166–171. doi: 10.1016/j.atherosclerosis.2016.03.034. 27084530

[19] Lanzer P, Hannan FM, Lanzer JD, et al. Medial arterial calcification: JACC state-of-the-art review. J Am Coll Cardiol. 2021;78(11):1145–1165. doi: 10.1016/j.jacc.2021.06.049. 34503684 PMC8439554

[20] Górriz JL, Molina P, Cerverón MJ, et al. Vascular calcification in patients with nondialysis CKD over 3 years. Clin J Am Soc Nephrol. 2015;10(4):654–666. doi: 10.2215/CJN.07450714. 25770175 PMC4386255

[21] Liu Y, Yang C, Yang X, et al. Prevalence and clinical characteristics of calciphylaxis in Chinese hemodialysis ­patients. Front Med. 2022;9:902171. doi: 10.3389/fmed.2022.902171. PMC9226545 35755071

[22] Chinnadurai R, Huckle A, Hegarty J, et al. Calciphylaxis in end-stage kidney disease: outcome data from the United Kingdom calciphylaxis study. J Nephrol. 2021;34(5):1537–1545. doi: 10.1007/s40620-020-00908-9. 33548054 PMC8494680

[23] Niu Q, Zhao H, Wu B, et al. Abdominal aortic calcification is superior to other arteries calcification in predicting the mortality in peritoneal dialysis patients – A 8 years cohort study. BMC Nephrol. 2019;20(1):439. doi: 10.1186/s12882-019-1593-6. 31791277 PMC6888938

[24] Niskanen L, Siitonen O, Suhonen M, et al. Medial artery calcification predicts cardiovascular mortality in patients with NIDDM. Diabetes Care. 1994;17(11):1252–1256. doi: 10.2337/diacare.17.11.1252. 7821163

[25] Kidney Disease: Improving Global Outcomes (KDIGO) CKD-MBD Update Work Group. KDIGO 2017 clinical practice guideline update for the diagnosis, evaluation, prevention, and treatment of chronic kidney disease-mineral and bone disorder (CKD-MBD). Kidney Int Suppl. 2017;7(1):1–59. doi: 10.1016/j.kisu.2017.04.001. PMC6340919 30675420

[26] Ikizler TA, Burrowes JD, Byham-Gray LD, et al. KDOQI clinical practice guideline for nutrition in CKD: 2020 update. Am J Kidney Dis. 2020;76(3 Suppl 1):S1–S107. doi: 10.1053/j.ajkd.2020.05.006. 32829751

[27] den Hoedt CH, Bots ML, Grooteman MP, et al. Clinical predictors of decline in nutritional parameters over time in ESRD. Clin J Am Soc Nephrol. 2014;9(2):318–325. doi: 10.2215/CJN.04470413. 24458074 PMC3913235

[28] Koppe L, Fouque D, Kalantar-Zadeh K. Kidney cachexia or protein-energy wasting in chronic kidney disease: facts and numbers. J Cachexia Sarcopenia Muscle. 2019;10(3):479–484. doi: 10.1002/jcsm.12421. 30977979 PMC6596400

[29] Fujioka H, Koike T, Imamura T, et al. Impact of geriatric nutritional risk index and modified creatinine index combination on mortality in hemodialysis patients. Nutrients. 2022;14(4):801. doi: 10.3390/nu14040801. 35215451 PMC8878210

[30] Couchoud C, Bayer F, Ayav C, et al. Low incidence of SARS-CoV-2, risk factors of mortality and the course of illness in the French national cohort of dialysis patients. Kidney Int. 2020;98(6):1519–1529. doi: 10.1016/j.kint.2020.07.042. 32858081 PMC7445552

[31] Doshi M, Streja E, Rhee CM, et al. Examining the robustness of the obesity paradox in maintenance hemodialysis patients: a marginal structural model analysis. Nephrol Dial Transplant. 2016;31(8):1310–1319. doi: 10.1093/ndt/gfv379. 26590266 PMC4967726

[32] Su G, Saglimbene V, Wong G, et al. Healthy lifestyle and mortality among adults receiving hemodialysis: the DIET-HD study. Am J Kidney Dis. 2022;79(5):688–698.e1. doi: 10.1053/j.ajkd.2021.07.022. 34547395

[33] Streja E, Kovesdy CP, Molnar MZ, et al. Role of nutritional status and inflammation in higher survival of African American and Hispanic hemodialysis patients. Am J Kidney Dis. 2011;57(6):883–893. doi: 10.1053/j.ajkd.2010.10.050. 21239093 PMC3081903

[34] Tanaka S, Nakano T, Hiyamuta H, et al. Impact of ­multivascular disease on cardiovascular mortality and morbidity in patients receiving hemodialysis: ten-year outcomes of the Q-Cohort study. J Atheroscler Thromb. 2021;28(4):385–395. doi: 10.5551/jat.54098. 32684556 PMC8147568

