# Supplementary material for: Characteristics and prognostic values of abdominal aortic branches calcification in hemodialysis patients
Source: Ren Fail. 2025 Jan 6;47(1):2432538. doi: 10.1080/0886022X.2024.2432538 (PMC11721613; doi:10.1080/0886022X.2024.2432538)
Supplement: revised_Supplementary_materials new.docx [file IRNF_A_2432538_SM8760.docx]

**Supplementary materials**

**Table 1.** Prevalence of abdominal vascular calcification.

| Calcified artery | *n* (%) |
| --- | --- |
| Abdominal aorta | 210 (88.61) |
| Celiac trunk | 151 (63.71) |
| mesenteric artery | 148 (62.45) |
| Renal artery | 171 (72.15) |
| Common iliac artery | 190 (80.17) |
| Internal iliac artery | 203 (85.65) |
| Branches of abdominal aortic artery | 224 (94.51) |
| Overall | 226 (95.36) |
| Only 1 artery | 7 (2.95) |
| 2 arteries | 13 (5.49) |
| 3 arteries | 31 (13.08) |
| 4 arteries | 33 (13.92) |
| 5 arteries | 37 (15.61) |
| All of the arteries | 105 (44.30) |
| None of the artery | 11 (4.64) |

**Table 2.** Comparison of causes of death according to total abdominal vascular calcification.

| Causes of death | Overall  (*N*=237) | Mild calcification (*n*=118) | Severe calcification  (*n*=119) | *P* value |
| --- | --- | --- | --- | --- |
| Cardiovascular event, *n* (%) | 74 (31.22) | 33 (27.97) | 41 (34.45) | 0.281 |
| Infection, *n* (%) | 26 (10.97) | 16 (13.56) | 10 (8.40) | 0.204 |
| Cancer, *n* (%) | 11 (4.64) | 5 (4.24) | 6 (5.04) | 0.768 |
| Gastrointestinal hemorrhage, *n* (%) | 7 (2.95) | 6 (5.08) | 1 (0.84) | 0.054 |
| Others, *n* (%) | 19 (8.02) | 12 (10.17) | 7 (5.88) | 0.224 |
| All-cause death, *n* (%) | 137 (57.81) | 51 (43.22) | 86 (72.27) | <0.001 |

**Table 3.** Univariate and multivariate Cox regression analysis assessing variables associated with survival in hemodialysis patients.

| Variables | Univariate analysis | | | Multivariate analysis | | |  |
| --- | --- | --- | --- | --- | --- | --- | --- |
|  | HR | 95%CI | *P*-value | HR | 95%CI | *P*-value |  |
| Age (year) | 1.05 | 1.03-1.07 | <0.001 | 1.03 | 1.01-1.05 | 0.002 |  |
| Male | 0.83 | 0.52-1.31 | 0.424 | - | - | - |  |
| Dialysis vintage (month) | 1.00 | 0.99-1.00 | 0.184 | - | - | - |  |
| BMI (kg/m^2^) |  |  | 0.057 |  |  | - |  |
| <18.50 | 1.77 | 0.95-3.31 | 0.072 | - | - | - |  |
| 18.50-23.99 | Reference | | | - | - | - |  |
| ≥24.00 | 0.75 | 0.44-1.30 | 0.307 | - | - | - |  |
| BSA (m^2^) | 0.13 | 0.03-0.49 | 0.003 | - | - | - |  |
| SBP (mmHg) | 1.00 | 0.99-1.01 | 0.580 | - | - | - |  |
| DBP (mmHg) | 0.97 | 0.95-0.99 | 0.001 | - | - | - |  |
| Smoking | 1.20 | 0.57-2.50 | 0.633 | - | - | - |  |
| Comorbidities |  |  |  |  |  |  |  |
| Hypertension | 0.60 | 0.34-1.06 | 0.077 | - | - | - |  |
| Cardiovascular disease | 2.40 | 1.51-3.82 | <0.001 | - | - | - |  |
| Atrial fibrillation | 1.40 | 0.78-2.51 | 0.256 | - | - | - |  |
| Parathyroidectomy | 0.04 | 0.00-1.1.38 | 0.075 | - | - | - |  |
| Medications |  |  |  |  |  |  | |
| Vitamin D and analogue | 0.94 | 0.59-1.50 | 0.784 | - | - | - | |
| Calcium-based phosphate binder | 0.87 | 0.49-1.57 | 0.65 | - | - | - | |
| Calcimimetic | 0.54 | 0.08-3.88 | 0.54 | - | - | - | |
| ACEI/ARB | 0.57 | 0.28-1.15 | 0.116 | - | - | - | |
| β-blocker | 0.68 | 0.42-1.09 | 0.11 | - | - | - | |
| Calcium channel blocker | 0.89 | 0.56-1.41 | 0.613 | - | - | - | |
| Warfarin | 0.05 | 0.00-190.331 | 0.473 | - | - | - | |
| Antiplatelet agent | 1.33 | 0.70-2.53 | 0.385 | - | - | - | |
| Nitrate | 2.05 | 1.27-3.32 | 0.003 | 1.86 | 1.13-3.07 | 0.015 | |
| Statin | 2.52 | 1.20-5.29 | 0.015 | - | - | - | |
| Hemoglobin (g/L) | 0.99 | 0.98-1.00 | 0.248 | - | - | - | |
| CRP (mg/L) | 1.01 | 1.00-1.01 | 0.02 | 1.01 | 1.00-1.01 | 0.005 | |
| Albumin (g/L) | 0.90 | 0.86-0.95 | <0.001 | 0.94 | 0.89-0.99 | 0.016 | |
| Alkaline phosphatase (U/L) | 1.00 | 1.00-1.01 | 0.028 | - | - | - | |
| Glucose (mmol/L) | 1.11 | 0.97-1.28 | 0.129 | - | - | - | |
| Creatinine (μmol/L) | 1.00 | 1.00-1.00 | 0.002 | - | - | - | |
| Uric acid (μmol/L) | 1.00 | 1.00-1.00 | 0.545 | - | - | - | |
| Triglyceride (mmol/L) | 1.18 | 0.93-1.49 | 0.169 | - | - | - | |
| Total cholesterol (mmol/L) | 1.17 | 0.95-1.43 | 0.133 | - | - | - | |
| HDL (mmol/L) | 1.72 | 0.67-4.41 | 0.262 | - | - | - | |
| LDL (mmol/L) | 1.04 | 0.81-1.33 | 0.767 | - | - | - | |
| Potassium (mmol/L) | 0.75 | 0.53-1.06 | 0.107 | - | - | - | |
| Corrected calcium (mmol/L) | 0.66 | 0.27-1.62 | 0.367 | - | - | - | |
| Phosphorus (mmol/L) | 0.92 | 0.66-1.27 | 0.602 | - | - | - | |
| Ferritin (μg/L) | 1.00 | 1.00-1.00 | 0.971 | - | - | - | |
| PTH (pg/ml) | 1.00 | 1.00-1.00 | 0.317 | - | - | - | |
| Arterial calcification volume (per 1SD log) |  |  |  |  |  |  | |
| Abdominal aorta | 1.30 | 1.08-1.57 | 0.006 | - | - | - | |
| Celiac trunk | 1.40 | 1.17-1.68 | <0.001 | - | - | - | |
| Mesenteric arteries | 1.61 | 1.31-1.98 | <0.001 | - | - | - | |
| Renal artery | 1.28 | 1.03-1.59 | 0.026 | - | - | - | |
| Common iliac artery | 1.37 | 1.13-1.66 | 0.002 | - | - | - | |
| Internal iliac artery | 1.67 | 1.34-2.07 | <0.001 | 1.39 | 1.10-1.76 | 0.006 | |

BMI, body mass index; BSA, body surface area; SBP, systolic blood pressure; DBP, diastolic blood pressure; ACEI, angiotensin-converting enzyme inhibitor; ARB, angiotensin receptor blocker; CRP, C-reactive protein; HDL, high density lipoprotein; LDL, low density lipoprotein; PTH, parathyroid hormone.

**Figure 1.** The screenshots of software 3D Slicer show the process of vascular calcification assessment. (a) Import images, and set the calcification threshold to over 130 Hounsfield units. Then paint the target artery, e.g., abdominal aorta, layer by layer. The areas marked in green on the CT image indicate calcification of abdominal aorta in a transverse section. (b) Transform two-dimensional images to three-dimensional models. The green model represents calcification throughout the abdominal aorta. The calcification volume can be calculated and displayed on the left side of the figure.

**Figure 2.** Composition of causes of death in deceased patients.

**Figure 3.** Kaplan-Meier cumulative incidence curves of death (a) and new-onset cardiovascular events (b) according to total abdominal vascular calcification.

**Figure 4.** Time-dependent ROC curves of abdominal vascular calcification and coronary artery calcification for predicting cardiovascular events.
